# Senescence in Monocytes Facilitates Dengue Virus Infection by Increasing Infectivity

**DOI:** 10.3389/fcimb.2020.00375

**Published:** 2020-07-28

**Authors:** Tzu-Han Hsieh, Tsung-Ting Tsai, Chia-Ling Chen, Ting-Jing Shen, Ming-Kai Jhan, Po-Chun Tseng, Chiou-Feng Lin

**Affiliations:** ^1^Department of Microbiology and Immunology, School of Medicine, College of Medicine, Taipei Medical University, Taipei, Taiwan; ^2^School of Respiratory Therapy, College of Medicine, Taipei Medical University, Taipei, Taiwan; ^3^Graduate Institute of Medical Sciences, College of Medicine, Taipei Medical University, Taipei, Taiwan; ^4^Core Laboratory of Immune Monitoring, Office of Research and Development, Taipei Medical University, Taipei, Taiwan; ^5^Center of Infectious Diseases and Signaling Research, National Cheng Kung University, Tainan, Taiwan

**Keywords:** dengue virus, senescence, IL-10, DC-SIGN, monocytes

## Abstract

Aging and chronic condition increase the incidence of dengue virus (DENV) infection, generally through a mechanism involving immunosenescence; however, the alternative effects of cellular senescence, which alters cell susceptibility to viral infection, remain unknown. Human monocytic THP-1 cells (ATCC TIB-202) treated with D-galactose to induce cellular senescence were susceptible to DENV infection. These senescent cells showed increased viral entry/binding, gene/protein expression, and dsRNA replication. The use of a replicon system showed that pharmacologically induced senescence did not enhance the effects on viral protein translation. By examining viral receptor expression, we found increased expression of CD209 (DC-SIGN) in the senescent cells. Interleukin (IL)-10 was aberrantly produced at high levels by the senescent cells, and the expression of the DENV receptor DC-SIGN was increased in these senescent cells, partially via IL-10-mediated regulation of the JAK2-STAT3 signaling pathway. The results demonstrate that a senescent phenotype facilitates DENV infection, probably by increasing DC-SIGN expression.

## Introduction

Mosquito-borne dengue virus (DENV), which is a positive-sense single-stranded RNA virus of the genus *Flavivirus*, causes infection in an estimated 50–100 million people annually (Guo et al., 2017). Infection with DENV causes dengue diseases, which range from mild dengue fever (DF) to more severe dengue hemorrhagic fever (DHF), dengue shock syndrome (DSS), central nervous system impairment, and multiorgan involvement (Guzman et al., 2016; Muller et al., 2017). In severe dengue, the exacerbated disease progression and increased case-fatality rate observed in patients have been associated with aging-related issues and common chronic diseases, such as allergies, diabetes, and hypertension (Wang et al., 2019). To obtain a better understanding of the complicated viral pathogenesis of DENV, several hypotheses, including the effects of viral load and virulence, the induction of cytotoxicity and pathological changes, and the immunopathogenesis of host immune alteration and autoimmunity, have been proposed (Diamond and Pierson, 2015; Guzman et al., 2016; Katzelnick et al., 2017). To improve dengue treatment and prevention, the development of safe and effective antiviral agents and vaccines is urgently needed.

During DENV infection, many host cells, including monocytes/macrophages, dendritic cells (DCs), B cells, T cells, basophil/mast cells, endothelial cells, epithelial cells, and hepatocytes, can be targeted by virions through different viral receptors (Cruz-Oliveira et al., 2015). Immune cells, such as monocytes/macrophages and DCs, that express dendritic cell-specific intracellular adhesion molecule 3 grabbing non-integrin (DC-SIGN), which is also known as CD209, are usually the target cells of DENV infection (Tassaneetrithep et al., 2003). The overexpression of DC-SIGN by cells increases the infectivity of DENV (Liu et al., 2017). After DC-SIGN-mediated infection, DENV-infected DCs may survive, become activated, and induce inflammation by increasing the production of multiple cytokines (Ho et al., 2001). Aberrant immune alterations, including immune cell activation, cytokine production, complement activation, inflammatory mediator production, antibody-dependent enhancement (ADE) of infection, and autoimmunity, have been proposed to participate in the immunopathogenesis of dengue (Martina, 2014; Begum et al., 2019).

Because older dengue patients accompanied by chronic condition have an elevated risk of developing severe multi-organ dysfunction/failure compared to younger dengue patients (Lin et al., 2017), there is an urgent need to explore the causes of severe dengue in susceptible individuals. In Singapore and Taiwan, susceptible cases tended to older adults are the main population at high risk of DENV infection, and this population suffers from severe disease burden (Rowe et al., 2014; Hsu et al., 2017; Wang et al., 2019). In gerontology, the reduction of host immunity and the induction of immunosenescence may cause older individuals to be susceptible to infection (Kline and Bowdish, 2016; Yao and Montgomery, 2016). Cellular senescence, particularly in cells of the immune system, is the hallmark of elderly adults (Salvioli et al., 2013; Nikolich-Zugich, 2018); however, the possibility of immunosenescence-enhanced DENV infection has not been explored. In this study, by using a chemical inducer of cellular senescence (Elzi et al., 2016), we examined the possible effects of D-galactose treatment on DENV infection in monocytes. The regulation of immunosenescence-enhanced DENV infection was also investigated.

## Materials and Methods

### Cells, Virus Strains, and Reagents

Cell lines used for this study, including human THP-1 monocytic cells (ATCC TIB-202), baby hamster kidney (BHK)-21 cells (ATCC, CCL10), and *Aedes albopictus* C6/36 cells (ATCC, CRL1660), were cultured by the standard procedures according to the previous works (Tsai et al., 2014). For cell culture, RPMI medium 1640 (RPMI; Invitrogen Life Technologies, Rockville, MD) and Dulbecco's modified Eagle's medium (DMEM; Invitrogen Life Technologies) were supplemented with 10% heat-inactivated fetal bovine serum (FBS; Invitrogen Life Technologies). A Taiwanese human isolated DENV2 PL046 was propagated in C6/36 cells and quantitated by using the BHK-21 cells. Reagents and antibodies used in these studies were as follows: D-galactose, 4,6-diamidino-2-phenylindole (DAPI), JAK2 inhibitor AG490, STAT3 inhibitor niclosamide, and a mouse monoclonal antibody (mAb) specific for β-actin (Sigma-Aldrich, St. Louis, MO); antibodies against dsRNA (English and Scientific Consulting, Szirák, Hungary); antibodies against p16, p21, p53, pSTAT3 (Tyr705), STAT3, SOCS3, and DC-SIGN (Cell Signaling Technology, Beverly, MA); antibodies against DENV NS1 (GeneTex, San Antonio, TX); neutralizing antibodies against DC-SIGN, IL-10, mouse IgG, and rabbit anti-mouse IgG conjugated with horseradish peroxidase (HRP) (Abcam, Cambridge, MA); and Alexa Fluor 488-conjugated goat anti-mouse antibodies (Invitrogen, Carlsbad, CA).

### Cell Viability and Cytotoxicity

For the detection of cell viability and cytotoxicity, a colorimetric Cell Counting Kit-8 (Dojindo Molecular Technologies, Kumamoto, Japan) and Cytotoxicity Detection kit assays (Roche Diagnostics, Lewes, UK) were respectively assessed according to the manufacturer's instructions. The concentration of formazan WST-8 [2-(2-methoxy-4-nitrophenyl)-3-(4-nitrophenyl)-5-(2,4-disulfophenyl)-2Htetrazolium,monosodium salt] and lactate dehydrogenase in each sample is then directly determined from the increase in absorbance at 460 and 340 nm, respectively, by using a microplate reader (SpectraMax 340PC; Molecular Devices Corporation, Sunnyvale, CA, USA).

### β-Galactosidase Activity

The cellular senescence of D-galactose-treated THP-1 cells was examined using a β-Galactosidase Detection Kit (Abcam) according to the manufacturer's instructions. The galactosidase induced cleavage of fluorogenic fluorescein digalactosidease can be detected with fluorescence instruments equipped with a fluorescein isothiocyanate filter set in spectra of excitation/emission = 490/515 nm. Images were observed and captured using a fluorescence microscope (BX51; Olympus, Tokyo, Japan), and the average relative fluorescence units (RFU) were calculated for all the samples.

### Cell Cycle Analysis

The cell cycle was analyzed using propidium iodide (PI; Sigma-Aldrich) staining, and then, the cells were analyzed using flow cytometry (FACSCalibur; BD Biosciences, San Jose, CA) with excitation at 488 nm and emission detected in the FL-2 channel (565–610 nm). The percentages of cells were analyzed by using CellQuest Pro 4.0.2 software (BD Biosciences).

### Western Blotting

Western blotting was performed according to our previous studies (Tsai et al., 2014; Kao et al., 2018). Briefly, harvested cells were lysed with a buffer and then centrifuged at 12,000 rpm at 4°C for 20 min. Lysates were boiled in sample buffer and then subjected to SDS-PAGE and transferred to PVDF membranes (Millipore, Billerica, MA, USA). After blocking with 5% skim milk in PBS, membranes were incubated with diluted primary antibodies and then incubated with diluted HRP-conjugated secondary antibodies for ECL development.

### Plaque Assay

Measurement of the viral titers was performed with a plaque assay according to previous studies (Tsai et al., 2014). Briefly, in DENV-infected BHK-21 cells, we fixed and stained the infected cellular monolayer by using 1% crystal violet (Sigma-Aldrich). The formation of plaques was counted in each well to calculate the titer of viral stock samples in terms of plaque forming units (pfu) per milliliter.

### Fluorescent DENV

DENV was labeled with Alexa Fluor 594 succinimidyl ester (AF594SE, Molecular Probes, Invitrogen) according to a previous study (Zhang et al., 2010). The labeled viruses were purified using Amicon Ultra filter (Millipore) to remove unbound dye and stored in 50 μl aliquots at −80 °C prior to use.

### Reverse-Transcription (RT)-Polymerase Chain Reaction (PCR) and Quantitative (q)PCR

A TRIZol (Invitrogen) RNA extraction reagent was used to extract total RNA, and a PrimeScriptTM RT reagent kit (Takara, Tokyo, Japan) was utilized to prepare complementary (c)DNA. Following a qPCR reaction conducted by using KAPA SYBR FAST qPCR Master Mix (Life Technologies and Kapa Biosystems, Woburn, MA), the PCR was performed using a StepOnePlusTM real-time PCR system (Applied Biosystems, Foster City, CA) with the following pair of specific primers: primer sequences for NS1 (forward): 5′-ATGGATCCGATAGTGGTTGCGTTGTGA-3′ and NS1 (reverse): 5′-ATCTCGAGGGCTGTGACCAAGGAGTT-3′.

### Immunostaining

Immunostaining of dsRNA was carried out according to our previous studies (Kao et al., 2018). Briefly, in fixed cells, anti-dsRNA antibodies were incubated overnight at 4°C followed by an incubation with secondary antibodies for 30 min. Cells were counterstained with 1 μg/ml DAPI in PBS for 5 min and washed three times with PBS. The cells were visualized under a fluorescence microscope (BX51; Olympus, Tokyo, Japan) or analyzed using flow cytometry (FACSCalibur). The mean fluorescence intensity (MFI) and the percentage of positive cells were analyzed by using CellQuest Pro 4.0.2 software.

### Reporter Assay

The viral protein translation activity was examined by using BHK-21 cells harboring the luciferase-expressing DENV replicon (BHK-D2-Fluc-SGR-Neo-1) according to a previous study (Kao et al., 2018). Briefly, cells were lysed and the luciferase activity was evaluated according to the manufacturer's instructions (Luciferase Assay System, Promega). The luciferase activity was measured by microplate reader (SpectraMax 340PC) at an absorption wavelength of 640 nm.

### ELISA

Expression of interleukin (IL)-10 was measured by using a commercial enzyme-linked immunosorbent assay (ELISA) kit (R&D, Minneapolis, MN) according to the manufacturer's instructions.

### Statistical Analysis

Data, presented as the mean ± standard deviation (SD), were analyzed by an unpaired Student's *t*-test or by one-way analysis of variance (ANOVA) with Tukey's multiple-comparison test. Statistical significance was defined as *p* < 0.05.

## Results

### Treatment With D-Galactose Induces Senescence and Enhances DENV Replication in Human Monocytic THP-1 Cells

**Figure 1:**
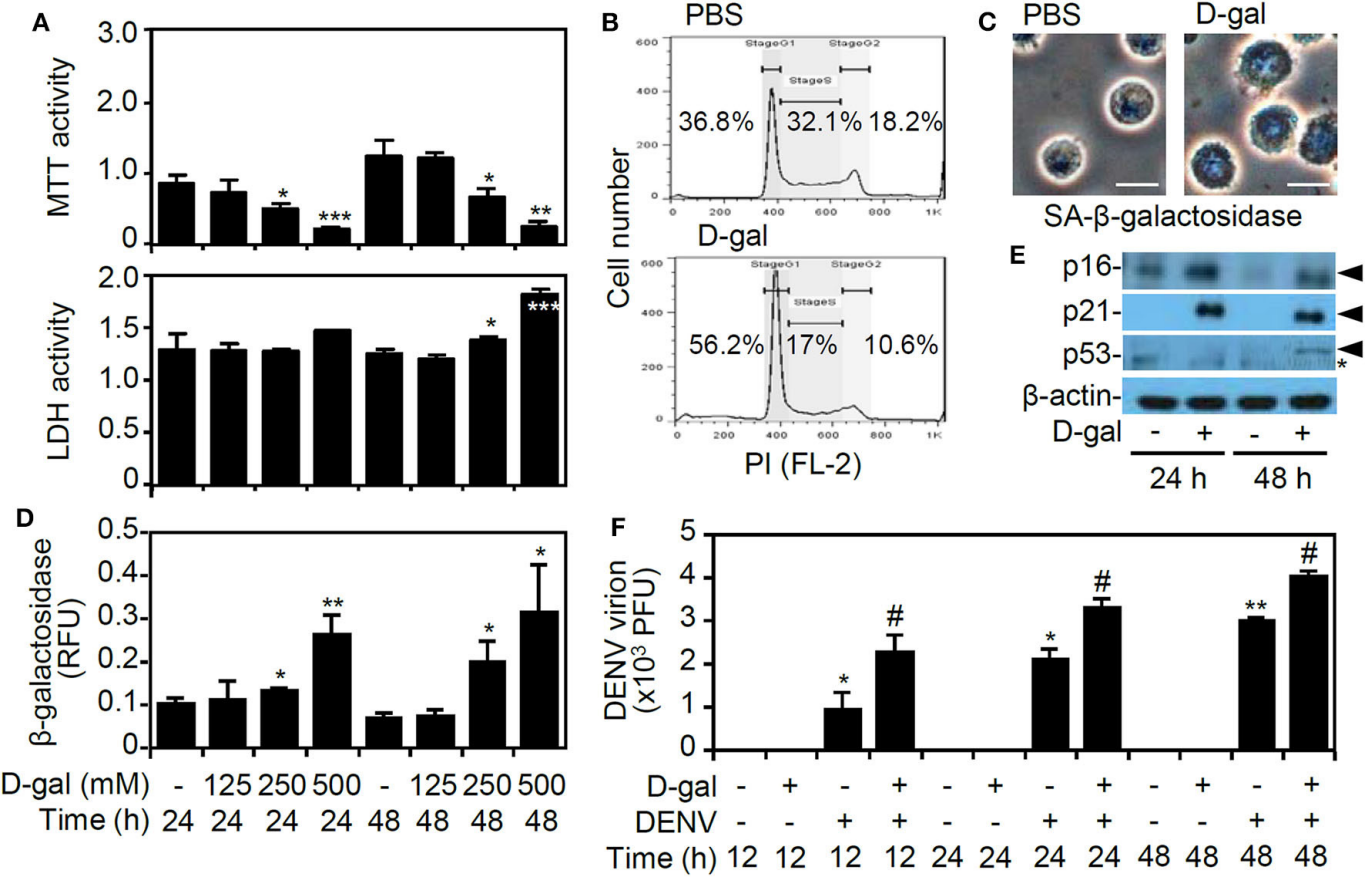
D-galactose treatment induces senescent responses and enhances dengue virus (DENV) serotype 2 PL046 (DENV2) replication in THP-1 cells. **(A)** The MTT and lactate dehydrogenase (LDH) assays showing cell viability and cytotoxicity in D-galactose (D-gal)-treated human monocytic THP-1 cells at the indicated doses and times. **(B)** Flow cytometry analysis showing the cell cycle phases in the D-gal (250 mM)-treated cells 48 hours poststimulation. A representative histogram and the percentages of cells are shown. **(C)** Microscopy analysis, **(D)** β-galactosidase activity assay, and **(E)** Western blot analysis of p16, p21, and p53 expression (arrowheads) showing senescent responses in D-gal (250 mM)-treated THP-1 cells. *, non-specific binding. **(F)** Plaque assays showing the level of viral replication in DENV2 (at a multiplicity of infection of 1)-infected THP-1 cells pretreated with or without D-gal (250 mM) for 48 hours. The quantitative data are shown as the mean ± SD. *p < 0.05, **p < 0.01, and ***p < 0.001, compared to the untreated cells. ^#^*p* < 0.05, compared to the DENV-infected cells.

In our previous studies, we established an *in vitro* model of DENV infection by using human monocytic THP-1 cells (Tsai et al., 2014, 2017). In this study, the monosaccharide sugar D-galactose was used to induce senescence (Elzi et al., 2016). MTT and LDH assays showed that D-galactose treatment significantly induced cell growth inhibition and cytotoxicity (*p* < 0.05) (Figure 1A). A propidium iodide-based flow cytometric analysis revealed the induction of cell cycle arrest at the G1 phase in the D-galactose-treated THP-1 cells (Figure 1B). Detection of the senescence-associated β-galactosidase activity by using imaging (Figure 1C) and activity assays (Figure 1D) demonstrated its significant senescent responses 48 h of D-galactose (250 mM) poststimulation. Additionally, following D-galactose treatment, the senescence-associated p16, p21, and p53 proteins, which are highly associated with growth arrest during senescence (Rufini et al., 2013), were increased (Figure 1E). A plaque assay showed that DENV caused significant infection of senescent THP-1 cells *in vitro* (*p* < 0.05) (Figure 1F). These results indicate the enhanced infection of DENV in senescent THP-1 cells.

### D-Galactose Treatment Facilitates DENV Infection by Enhancing Viral Entry, Viral Gene/Protein Expression, and dsRNA Replication in THP-1 Cells

**Figure 2:**
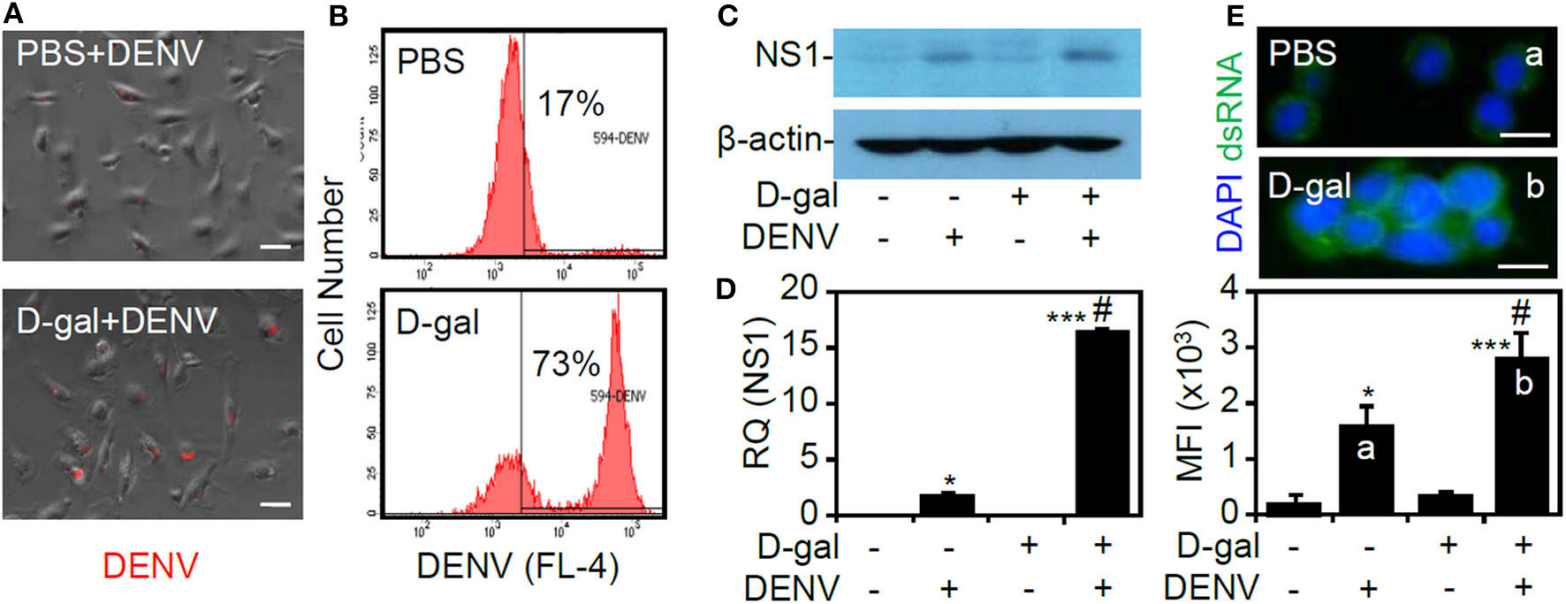
D-galactose treatment enhances dengue virus (DENV) serotype 2 PL046 (DENV2) infection, including viral entry, viral gene/protein expression, and double-stranded (ds)RNA replication in THP-1 cells. **(A)** Fluorescence microscopy and **(B)** flow cytometry were used to measure the expression and percentage of THP-1 cells infected with fluorescent Alexa-594-labeled DENV2 (at a multiplicity of infection of 1) for 2 h pretreated with or without D-galactose (D-gal; 250 mM). In D-gal-stimulated THP-1 cells 48 h posttreatment followed by DENV infection for further 24 h, **(C)** representative Western blot analysis showing viral non-structural protein 1 (*NS1*) expression. β-actin was used as an internal control. **(D)** In addition, qPCR showing the mRNA expression of NS1. **(E)** Immunocytochemistry and the relative mean fluorescence intensity (*MFI*) of viral dsRNA (*green*) in D-gal-stimulated THP-1 cells 48 h posttreatment followed by DENV infection for further 6 h. DAPI staining indicates nuclei (*blue*). For all the images, the representative data were selectively obtained from three individual experiments. The quantitative data are shown as the mean ± SD. *p < 0.05 and ***p < 0.001, compared to the untreated cells. ^#^*p* < 0.05, compared to the DENV-infected cells.

To investigate the possible effects of the senescent responses on facilitating DENV infection, the infectious steps of the viral life cycle, including binding/entry, viral protein expression, and dsRNA replication, were assessed (Rodenhuis-Zybert et al., 2010). By studying infection with fluorescently labeled DENV by fluorescence microscopy (Zhang et al., 2010), enhanced viral binding/entry could be identified in the D-galactose-treated THP-1 cells (Figure 2A). Furthermore, flow cytometric analysis revealed an increased percentage of cells infected with DENV2 in the senescent group (Figure 2B). Following binding/entry, Western blot analysis (Figure 2C) and quantitative PCR (Figure 2D) showed significantly increased expression of the viral NS1 protein and gene at 24 h postinfection (*p* < 0.05). Furthermore, immunostaining of dsRNA at 6 h postinfection showed that the senescent response significantly (*p* < 0.05) enhanced viral dsRNA replication (Figure 2E). The data confirm that senescent conditions enhance DENV infection in THP-1 cells.

**Figure 3:**
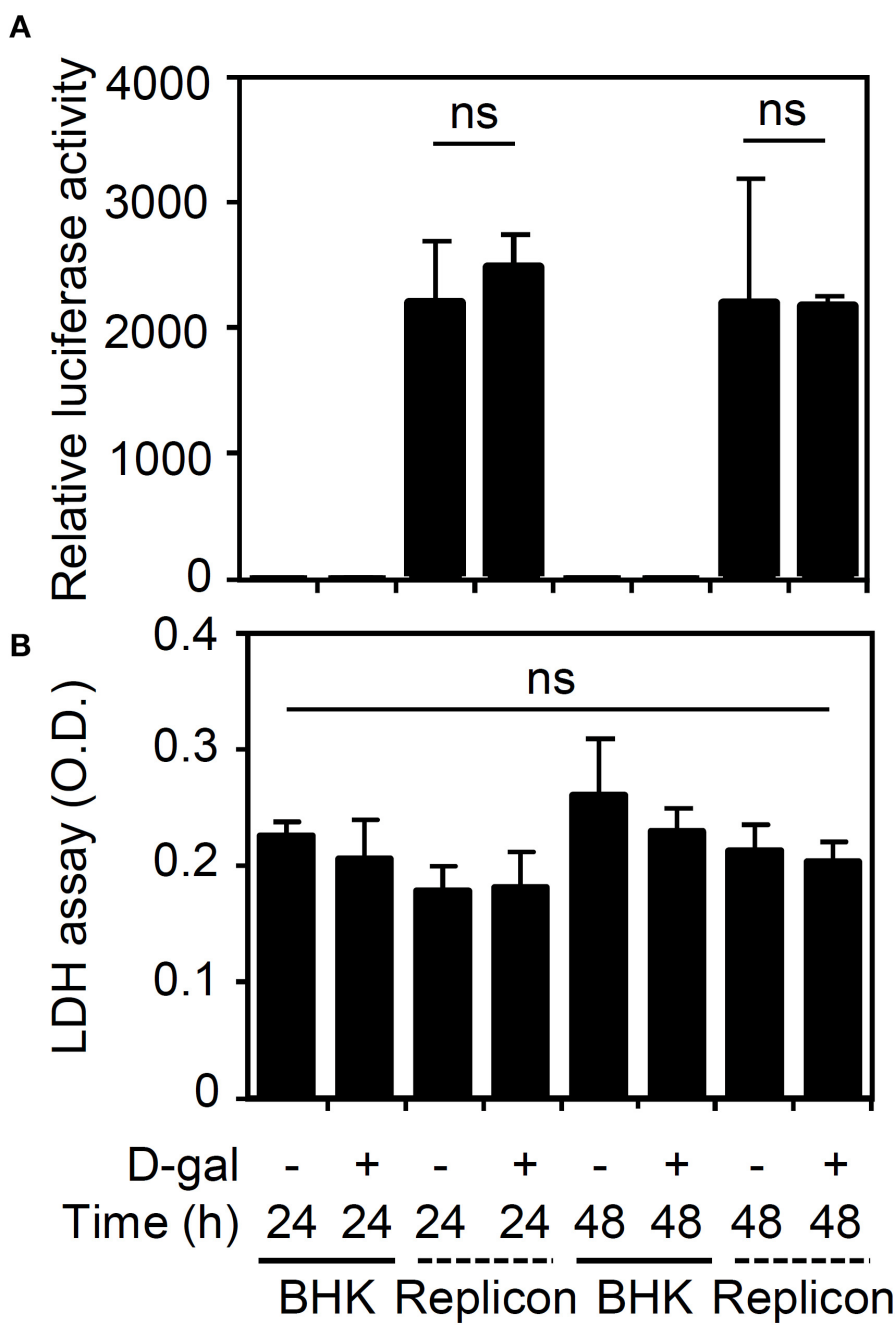
Treatment with D-galactose does not repress firefly luciferase activity in BHK-D2-Fluc-SGR-Neo-1 cells. **(A)** Luciferase activity and **(B)** lactate dehydrogenase (LDH) assays in D-galactose (D-gal; 250 mM)-treated parental BHK-21 and BHK-D2-Fluc-SGR-Neo-1 cells (replicons) 24 and 48 h posttreatment. The quantitative data are shown as the mean ± SD of three independent experiments. ns, not significant.

To further validate the effects of the senescent response on enhancing viral infection, we next examined the effects of senescence on viral protein translation by measuring firefly luciferase activity in BHK-D2-Fluc-SGR-Neo-1 cells. We found that treatment with D-galactose caused no inhibitory effects on viral translation (Figure 3A) or cytotoxicity in the cells (Figure 3B). These results indicate that the senescent response facilitates DENV infection by a mechanism that is independent of the direct promotion of viral protein translation.

### Induction of Senescence by D-Galactose Causes Increased Expression of DC-SIGN in THP-1 Cells

**Figure 4:**
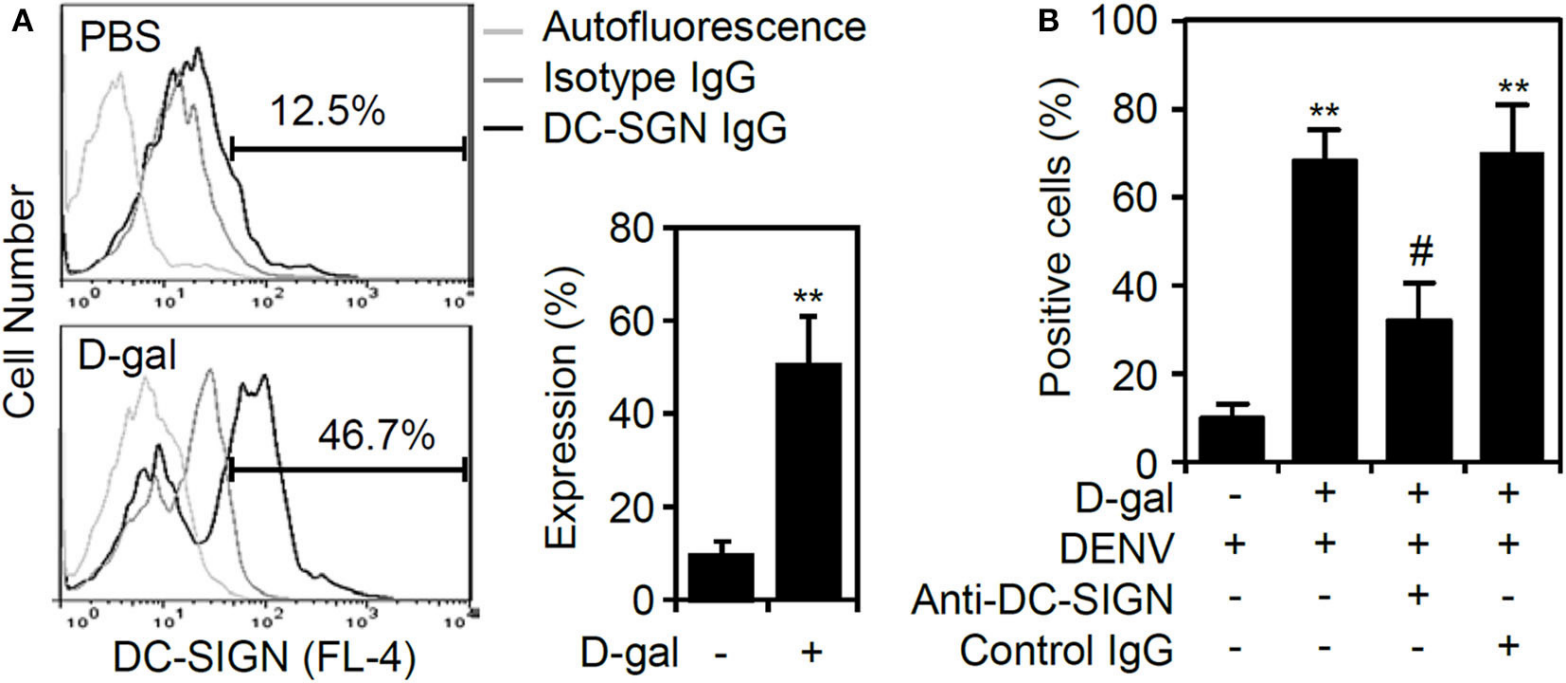
Increased expression of DC-SIGN in D-galactose-treated THP-1 cells. **(A)** Immunostaining followed by flow cytometric analysis showing the expression of DC-SIGN in the D-gal-stimulated THP-1 cells 48 h posttreatment. A representative histogram and the percentage of positive cells are shown, and the data are the mean ± SD of three independent experiments. **(B)** Flow cytometry measured the infectivity of fluorescent Alexa-594-labeled DENV2 (at a multiplicity of infection of 1) 2 h post-infection in D-galactose (D-gal; 250 mM)-treated THP-1 cells in the presence and absence of neutralizing anti-DC-SIGN and control IgG (5 μg/ml). **p < 0.01 compared to the DENV-infected cells. ^#^*p* < 0.05, compared to the DENV-infected senescent cells.

According to our results that the senescent response facilitated DENV binding/entry to enhance viral replication, we next examined the possible effects of D-galactose on the expression of the DENV receptor. Notably, THP-1 cells become susceptible to DENV infection in a DC-specific ICAM-3 grabbing non-integrin (DC-SIGN)-mediated manner (Tassaneetrithep et al., 2003). Senescent primary CD14^+^CD16^+^ monocytes exhibit increased expression of DC-SIGN and become further activated (Merino et al., 2011). The results of immunostaining followed by flow cytometric analysis demonstrated a significant increase (*p* < 0.01) in DC-SIGN expression in senescent THP-1 cells (Figure 4A). Treatment of neutralizing antibody against DC-SIGN significantly (*p* < 0.05) reversed D-galactose-enhanced DENV infectivity (Figure 4B). The findings reveal that the senescent response in monocytes causes the increased expression of DC-SIGN to facilitate DENV infection.

### Expression of IL-10 Is Increased in Senescent THP-1 Cell, and Pharmacologically Targeting the IL-10-JAK2-STAT3 Signaling Pathway Eliminate DC-SIGN Expression and DENV Infectivity

**Figure 5:**
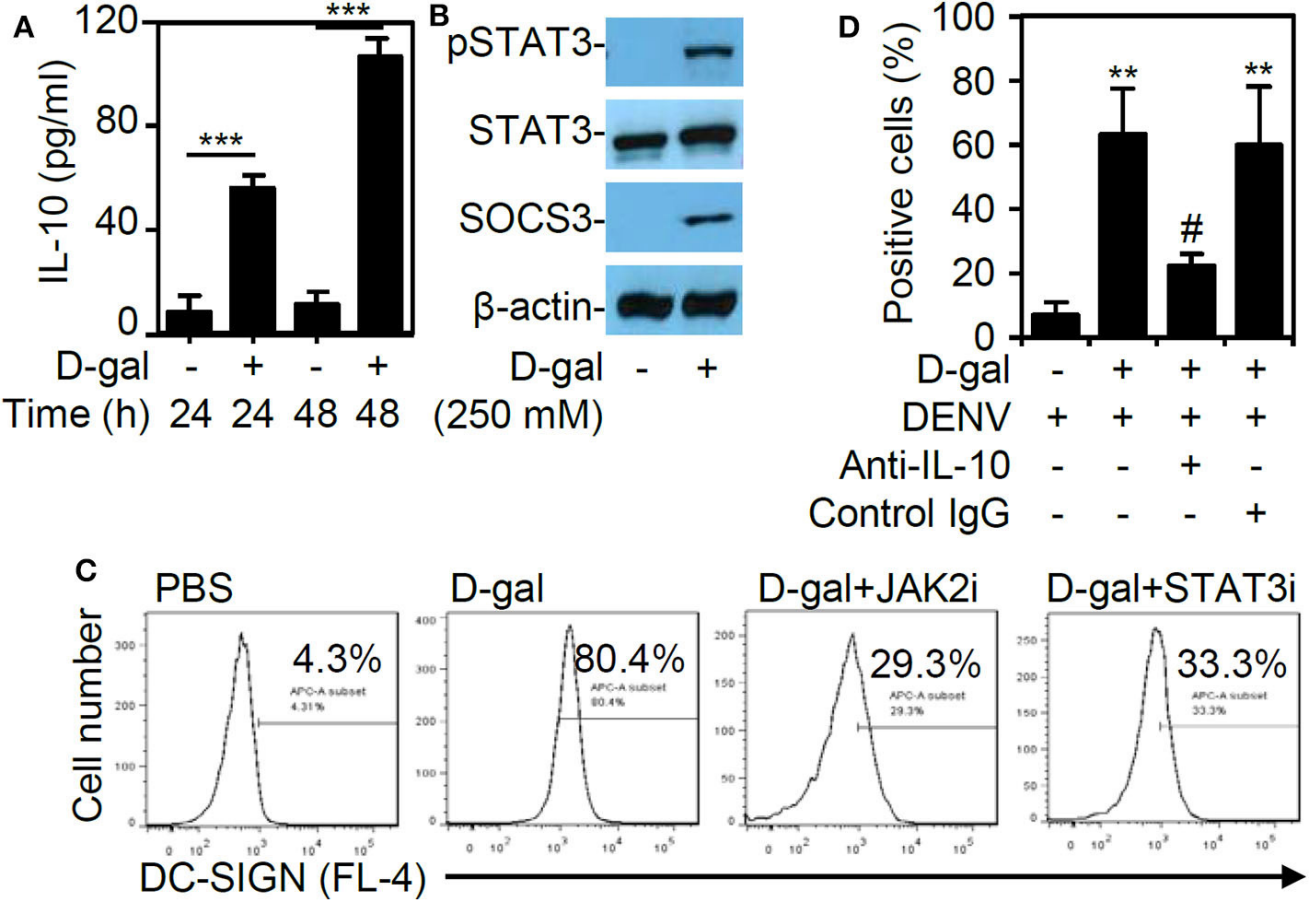
D-galactose treatment induces IL-10 production and activation followed by JAK2-STAT3-regulated DC-SIGN expression. **(A)** ELISA data showing the IL-10 expression in the THP-1 cells treated with D-galactose (D-gal; 250 mM). The quantitative data are shown as the mean ± SD of three independent experiments. ***p < 0.001. **(B)** Representative Western blot analysis of pSTAT3 (Tyr705), STAT3, and SOCS3 protein expression 48 h poststimulation. β-actin was used as an internal control. **(C)** Cotreatment with or without the JAK2 inhibitor AG490 (JAK2i; 100 μM) and the STAT3 inhibitor niclosamide (SATA3i; 0.1 μM), immunostaining followed by flow cytometric analysis showing the expression of DC-SIGN in the D-gal-stimulated THP-1 cells. A representative histogram and the percentage of positive cells are shown. **(D)** Flow cytometry measured the infectivity of fluorescent Alexa-594-labeled DENV2 (at a multiplicity of infection of 1) 2 h post-infection in D-galactose (D-gal; 250 mM)-treated THP-1 cells in the presence and absence of neutralizing anti-IL-10 and control IgG (5 μg/ml). **p < 0.01 compared to the DENV-infected cells. ^#^*p* < 0.05, compared to the DENV-infected senescent cells.

In this study, the chemical D-galactose was used to induce senescence in THP-1 cells. Because the cytokine IL-10 has been utilized to trigger senescence in primary CD14^+^CD16^+^ monocytes (Merino et al., 2011), we next examined the involvement of IL-10 in promoting THP-1 cell senescence following D-galactose treatment. First, the ELISA results revealed a significant and time-dependent increase (*p* < 0.001) in IL-10 expression in the THP-1 cells treated with D-galactose (Figure 5A). Western blot analysis further demonstrated the activation of signaling molecules downstream of IL-10, including the phosphorylation of STAT3 at Tyr705 and SOCS3, in the senescent THP-1 cells 48 h poststimulation (Figure 5B). To verify the role of IL-10-induced JAK2-STAT3 signaling in modulating DC-SIGN expression, the cells were treated with the JAK2 inhibitor AG490 and the STAT3 inhibitor niclosamide. These inhibitors effectively blocked the D-galactose-induced DC-SIGN expression, as demonstrated by immunostaining followed by flow cytometric analysis (Figure 5C). Exogenous treatment of anti-IL-10 neutralizing antibody significantly (*p* < 0.05) reversed D-galactose-enhanced DENV infectivity (Figure 5D). These results confirm that DC-SIGN expression is induced in senescent THP-1 cells via IL-10-mediated regulation of the JAK2-STAT3 signaling pathway.

## Discussion

**Figure 6:**
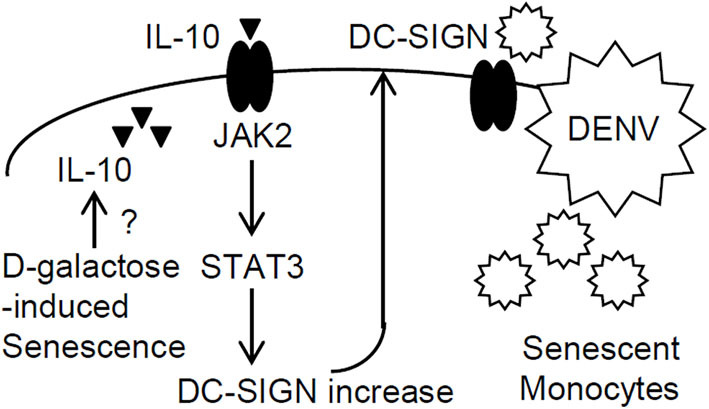
A hypothetical model of enhanced DENV infection in senescent monocytes. In this study, the chemical D-galactose-induced senescent response in monocytic THP-1 cells caused IL-10 production through an unknown mechanism. Then, IL-10 triggered JAK2-STAT3 signaling-regulated DC-SIGN expression to enhance DENV infection.

The present work investigated the possible mechanisms of the association between cellular senescence and the risk of DENV infection and demonstrated, for the first time, that senescence enhances DENV infection *in vitro*. Treatment of D-galactose may induce senescence in monocytes/macrophages *in vitro* (Zhang et al., 2019) and naturally aged mice (20–22-month-old) present accumulated senescent macrophages *in vivo* (Cai et al., 2020). Based on these findings, it is important to validate the increased infectivity of DENV in senescent primary monocytes/macrophages with increased DC-SIGN expression in older adults or aged mice in future studies. As summarized in Figure 6, the results showed that the chemical sugar D-galactose efficiently induced cellular senescence in human monocytic THP-1 cells. Notably, the senescent THP-1 cells were susceptible to DENV infection, indicating that senescence enhanced the infection process. Through the induction of the IL-10-activated JAK2-STAT3 signaling pathway, senescence promoted the increased expression of DC-SIGN to facilitate the infectivity of DENV.

In the elderly, immune cell senescence has been linked to poor responses to infections, vaccines, and immune therapy and has been associated with chronic low-grade inflammation, which increases morbidity and mortality (Salvioli et al., 2013; Nikolich-Zugich, 2018; Oh et al., 2019). Through the immune profiling of isolated classical monocytes from younger and older adults, the number of circulating classical monocytes is reduced in the older population, while intermediate and non-classical monocytes are increased with age (Pence and Yarbro, 2018). Notably, senescent intermediate monocytes exhibit proinflammatory activity, which is highly associated with chronic inflammatory disorders (Hanai et al., 2008; Merino et al., 2011). Until now, no further reports have shown the infectivity of DENV in senescent monocytes. However, DENV infects primary monocytes *in vitro* and induces cell death to trigger a pro-inflammatory response (Tan and Chu, 2013). We showed that chemical induction of cellular senescence increases DENV infection in THP-1 cells *in vitro*. It is speculated that an increased infectious rate is observed in older adults due to the presence of senescent monocytes.

During immunosenescence, resistance and a decreased antiviral interferon response have been demonstrated (Molony et al., 2018; Oh et al., 2019). Therefore, the lack of immune defense renders senescent cells susceptible to viral infection. The expression of immune sensors, such as Toll-like receptors (TLRs) and cytoplasmic retinoic acid-inducible gene I-like receptors, is poorly upregulated in response to pathogens. However, there are no further investigations on the effect of cellular senescent responses on routes of infection. In this study, we provided evidence to show that the induction of DC-SIGN facilitates DENV infection in senescent THP-1 cells. As a main viral receptor that mediates DENV binding to/entry into DCs and some specialized/differentiated monocytes/macrophages (Tassaneetrithep et al., 2003), the findings of this study indicate that senescence enhances DENV infection in monocytes in a DC-SIGN-mediated manner. Additionally, it is speculated that increased DC-SIGN expression in senescent monocytes may also facilitate other microbial infections, including infections with viruses such as influenza virus, human immunodeficiency virus, Ebola virus, hepatitis C virus, cytomegalovirus, and SARS coronavirus; infections with bacteria such as *Mycobacterium tuberculosis* and *Helicobacter pylori*; and infections with parasites such as *Leishmania pifanoi*, and that these pathogens need DC-SIGN as an infectious receptor (Khoo et al., 2008). Notably, most of the infections with these pathogens are observed in older adults.

The regulation of DC-SIGN expression is multifaceted. In response to TLR activation, the bacterial endotoxin lipopolysaccharide causes bone marrow monocyte differentiation into DCs, which are characterized by increased the expression of CD80, CD86, and DC-SIGN (Cheong et al., 2010). In addition, stimulation by the helper T cell cytokines IL-4, IL-10, and IL-13 upregulates DC-SIGN expression on monocyte-derived macrophages and DCs (Relloso et al., 2002; Soilleux et al., 2002; Merino et al., 2011). Aberrant IL-10 expression has been demonstrated to be pathogenic in DENV infection by facilitating DENV replication following an immunosuppressive mechanism (Malavige et al., 2013; Tsai et al., 2013). In D-galactose-treated THP-1 cells, IL-10 is unexpectedly overproduced and triggers the JAK2-STAT3 signaling pathway to regulate DC-SIGN expression. Although the mechanism of IL-10 induction by D-galactose in THP-1 cells remains unknown, these findings not only confirm the regulation of DC-SIGN by IL-10 but also reveal an effect of senescence on enhancing DC-SIGN-mediated DENV infection. Furthermore, the induction of IL-10 by senescent stress may also confer promoting effects on facilitating infectivity not only in increasing DC-SIGN in monocytes/macrophages but also in causing immunosuppressive effects scape from antiviral immunity both in immune and non-immune cells.

Senescence is present not only in older adults but also in chronic disorders, and it is a key factor that facilitates microbial infection (Salvioli et al., 2013). In addition to older adults, patients with several chronic underlying diseases are also susceptible to DENV infection (Hsu et al., 2017; Wang et al., 2019). Additionally, several hyperendemic countries in South America and Southeast Asia, an increased infectivity in non-aged patients show the involvement of environmental conditions and immunopathogenesis (Campos et al., 2015; Phanitchat et al., 2019; Bavia et al., 2020). Both in aged and non-aged population, senescent response may also distress immunopathogenesis of DENV infection on extrinsic ADE by affecting Fc receptor expression and signaling as well as on intrinsic ADE by enhancing IL-10 production and activation (Halstead et al., 2010). Either in dengue patients or in DENV-susceptible people, who are aging and chronic diseases' population, the presence of senescent monocytes/macrophages must be examined to validate their pathogenic role in facilitating DENV infection. In conclusion, based on the findings of this study, senescent monocytes aberrantly express DC-SIGN to increase the infectivity of DENV. It is thought that IL-10 expression and activation regulate the DC-SIGN expression that facilitates DENV infection. Targeting senescence and IL-10 may diminish DENV-susceptible monocytes and may be an approach for future anti-dengue therapy.

## Data Availability Statement

All datasets generated for this study are included in the article/supplementary material.

## Author Contributions

T-HH and T-TT performed most of the experiments and interpreted the results. C-LC and C-FL participated in the design and supervision of the projects. T-JS and M-KJ conducted the virus experiments. P-CT contributed to flow cytometry analysis. T-HH, T-TT, and C-FL designed the concept of the project and wrote the manuscript. All authors reviewed and approved the manuscript.

## Conflict of Interest

The authors declare that the research was conducted in the absence of any commercial or financial relationships that could be construed as a potential conflict of interest.
